# The carbon and nitrogen ecophysiologies of two endemic tropical orchids mirrors those of their temperate relatives and the local environment

**DOI:** 10.1098/rsos.160427

**Published:** 2016-11-23

**Authors:** Nicole A. Hynson

**Affiliations:** Department of Botany, University of Hawaii Manoa, 3190 Maile Way Room 101, Honolulu, HI 96822, USA

**Keywords:** Orchidaceae, tropical ecology, Hawaii, stable isotopes, mycorrhiza, rhizoctonia

## Abstract

Orchids are one of the most widely distributed plant families. However, current research on the ecophysiology of terrestrial orchids is biased towards temperate species. Thus, it is currently unknown whether tropical terrestrial orchids belong to similar trophic guilds as their temperate relatives. To examine the ecophysiologies of two tropical terrestrial orchids, I analysed the carbon and nitrogen stable isotope compositions and nitrogen concentrations of the Hawaiian endemics *Anoectochilus sandvicensis* and *Liparis hawaiensis*. I compared these values with those of surrounding vegetation and their temperate relatives. I found that *A. sandvicensis* was consistently enriched in the heavy isotope of nitrogen (^15^N) and had higher nitrogen (N) concentrations than surrounding vegetation, and these values were even higher than those of its temperate relatives. Carbon stable isotope composition among populations of *A. sandvicensis* varied by island. These results point to local environment and evolutionary history determining the ecophysiology of this species. Whereas *L.hawaiensis* was also enriched in ^15^N and had on average higher N concentrations than surrounding vegetation, these values were not significantly different from temperate relatives, indicating that evolutionary history may be a stronger predictor of this orchid species' ecophysiology than environment. I suggest that both Hawaiian species are potentially partially mycoheterotrophic.

## 1. Introduction

Orchids are an example of one of the most successful colonizations of the Earth by a single plant family, they exist on every continent and their distributions extend to even the most remote oceanic islands. Orchidaceae is also one of the most species-rich plant families and contains a diversity of functional guilds from epiphytes, to terrestrial herbs and evergreen species [1]. Perhaps one of the most interesting guilds of orchids is the non-photosynthetic mycoheterotrophs [2]. Mycoheterotrophy entails plants meeting all, or a portion of their carbon demands via symbiotic interactions with root-inhabiting fungi [3]. All orchids studied to date are considered initially mycoheterotrophic as they rely on fungi to provide germinating seeds with the necessary carbohydrates and nutrients for development [4]. Asorchids mature, their dependency on fungal nutrition ranges from full to partial mycoheterotrophy to autotrophy [5].

Detection of partial mycoheterotrophy among orchids poses a particular challenge, as these are leafy green individuals that based on appearance, generally look just like their autotrophic relatives. Two lines of evidence have been used to establish whether an orchid is partially mycoheterotrophic. The first is the analysis of the naturally abundant carbon and nitrogen stable isotope profiles of orchids. Many partially mycoheterotrophic species are enriched in both the heavy isotope of carbon (^13^C) and nitrogen (^15^N) relative to surrounding autotrophic species [6]. In the case of carbon (C), this enrichment is owed to the use of fungal-derived compounds for growth and reproduction, rather than C assimilated through photosynthesis [6]. The cause of ^15^N enrichment among partially mycoheterotrophic species is less clear, but often mirrors that of fully mycoheterotrophic species and may be owed to the use of organic substrates originating from fungi, rather than assimilated from the soil [6]. The second line of evidence for partial mycoheterotrophy has been molecular identification of plants' mycorrhizal fungal symbionts. Originally, Bidartondo *et al*. [7] found that some leafy green orchid species were both enriched in ^13^C and ^15^N relative to other species, and partnered with ectomycorrhizal fungi [7]. Ectomycorrhizal fungi had already been established as the hosts to fully mycoheterotrophic orchids [8], thus it was proposed that partially mycoheterotrophic species must also solely rely on this guild of fungi [9]. However, more recent analyses have revealed that partial mycoheterotrophy is much more widespread within Orchidaceae than previously thought [10]. The majority of orchid species associate with the polyphyletic group of orchid mycorrhizal fungi known as the rhizoctonias [4]. These fungi include Tulasnellaceae, Ceratobasidiaceae and Sebacinales species that range in their ecologies from saprotrophs, to endophytes, to mycorrhizal with other plant groups [4]. In the case of rhizoctonia-associated orchids, carbon stable isotope profiles do not always provide clear evidence of partial mycoheterotrophy [10–13]. However, recent measurements of additional ecophysiological traits has led researchers to question whether some species that are not enriched in ^13^C, but significantly enriched in ^15^N may be at least partially reliant on fungal nutrition [10].

Suspicion of partial mycoheterotrophy in rhizoctonia-associated orchids arose when researchers were consistently finding significant ^15^N enrichment, and in some cases significantly higher leaf nitrogen content, relative to other orchids and surrounding autotrophs [6]. Initially, these species were referred to as cryptic mycoheterotrophs [6]. More recently, Gebauer *et al*. [10] hypothesized that enrichment in hydrogen stable isotopes can be used as a proxy for carbon gains via fungi, as all organic compounds exchanged between fungi and orchids contain hydrogen and carbon [10]. Analysing the stable isotope profiles of deuterium (^2^H) and oxygen (^18^O) they found that four species of rhizoctonia-associated orchids are similarly enriched in these isotopes as ectomycorrhizal partially and fully mycoheterotrophic orchid species. These findings support the widespread existence of partial mycoheterotrophy among rhizoctonia-associated orchids, and that significant ^15^N enrichment can be used as an indicator of partial mycoheterotrophy. However, previous studies of rhizoctonia-associated orchids have all been carried out in temperate or Mediterranean climates [7,11–19] so it remains to be seen if the patterns of ^15^N enrichment and high leaf nitrogen content hold for related tropical species.

To date the stable isotope profiles of tropical orchids have only been examined for epiphytic crassulacean acid metabolism (CAM) species [20] and terrestrial fully mycoheterotrophic species associating with either saprotrophic [21–23] or ectomycorrhizal [23,24] fungi. In contrast to mycoheterotrophic species, CAM epiphytic orchids tend to be depleted in ^15^N and enriched in ^13^C and have lower leaf N content relative to their host trees [20]. Whereas similar to their temperate counterparts, tropical fully mycoheterotrophic orchids associated with ectomycorrhizal or saprotrophic fungi are enriched in both ^13^C and ^15^N, and have higher N content than surrounding autotrophs [6,22–24].

Terrestrial rhizoctonia-associated orchids are common in the tropics [25], but their stable isotope profiles and nitrogen content have yet to be examined, thus it remains to be seen whether ecophysiological patterns of these orchids remain consistent across climatic zones. Though microhabitats vary greatly within the tropics, due to generally greater nitrogen availability in the tropics relative to more temperate regions, one might predict that tropical species would be more enriched in ^15^N (owing to preferential loss of ^14^N via leaching and denitrification) and have overall greater leaf nitrogen content [26]. If this were the case, then any relative enrichment in ^15^N or relatively higher N content owed to partial mycoheterotrophy in tropical rhizoctonia-associated orchids may be masked [6,10]. Alternatively, tropical rhizoctonia-associated orchids that are capable of partial mycoheterotrophy may be more enriched in ^15^N and have nitrogen contents that are significantly higher than both surrounding autotrophic vegetation and those of their temperate relatives. In this study, I set out to examine the carbon and nitrogen ecophysiologies of two tropical orchid species endemic to the Hawaiian Islands.

The Hawaiian Islands are host to three native orchid species, which are also endemic to the islands, *Anoectochilus sandvicensis* (tribe Cranichideae), *Liparis hawaiensis* (tribe Malaxideae) and the critically endangered species *Peristylus holochila* (= *Platanthera holochila* tribe Orchideae) [27,28]. Owing to the rarity of *P. holochila*, I focused this study only on *L. hawaiensis* and *A. sandvicensis*. Both species are found on all the major Hawaiian Islands [27], but are considered rare with *A. sandvicensis* classified as a vulnerable species by the IUCN Red List of Threatened Species [29]. The genus *Liparis* has a global distribution with the exception of the poles. It is estimated to have over 400 species and is well represented throughout the Pacific Islands and particularly rich in the Palaeotropics [30]. The genus *Anoectochilus* has more than 40 species and is widespread throughout tropical regions [31].

Tribal relatives of *A. sandvicensis* and *L. hawaiensis* vary in their mycorrhizal specificity, from associating with a single clade or species of *Tulasnella* in *Goodyera pubescens* and *Liparis liliifolia*, respectively [32], to more broad associations with a diversity of *Ceratobasium* and *Tulasnella* species in *A. formosanus* [31] and *L. loeselii* [33]. The sister species to *L. hawaiensis*, *L. japonica* associates only with specific *Tulasnella* species [34], while other species of *Anoectochilus* appear to be more diverse in their fungal partnerships associating with rhizoctonias and in *A. roxburghii*, at least one normally saprotrophic species of *Mycena* [31,35]. Based on Sanger sequencing of the fungal barcode locus (the nuclear ribosomal internal transcribed spacer region) from a subset of the individuals of *A. sandvicensis* and all individuals of *L. hawaiensis* from the current study both species associate strictly with either *Ceratobasidium* spp. or *Tulasnella* spp., respectively (N.A.H. 2016, unpublished data). Furthermore, there is only one currently identified native ectomycorrhizal host tree in Hawaii (*Pisonia sandwicensis*), and its distribution does not overlap with the endemic orchids [36]. Combined, these lines of evidence support *L. hawaiensis* and *A.sandvicensis* belonging to the rhizoctonia-associated guild of orchids.

In this study, I address the following questions: (i) based on their ^15^N and ^13^C compositions and N concentrations do two tropical terrestrial orchid species show indications of partial mycoheterotrophy? (ii) Do these orchids exhibit similar patterns of ^15^N composition, and N concentrations as their temperate relatives? (iii) Do these tropical orchids and their temperate relatives have similar patterns of ^15^N and ^13^C enrichment relative to their respective surrounding vegetation? To address these questions, I compare the isotope profiles and nitrogen concentrations of *A. sandvicensis* and *L. hawaiensis* to neighbouring plants as well as their temperate orchid relatives.

## 2. Material and methods

### 2.1. Sampling scheme and site descriptions

Over a 2-year period from July 2013 to July 2015, I sampled 11 sites across four islands (Hawaii, Maui, Oahu and Kauai) containing the Hawaiian orchid *Anoectochilus sandvicensis* and three sites from Oahu with the orchid *Liparis hawaiensis* (table 1). Populations of the two orchid species are rare within the islands, but can be locally abundant. *Anoectochilus sandvicensis* is a terrestrial creeping herb with extensive rhizomes, while *L. hawaiensis* is a terrestrial or epiphytic herb with rooting rhizomes and pseudobulbs from which the leaves arise [27]. Both species are generally confined to native rainforest habitats and are frequently rooted in humus, decaying logs or in the case of *L. hawaiensis* growing epiphytically. Historically *A. sandvicensis* occurred between 300 and 1500 m in elevation, and *L. hawaiensis* could be found from sea level to over 3000 m in elevation [27]. Today, populations of both orchids are primarily restricted to higher elevation sites where native vegetation remains intact (Hank Oppenheimer 2015, personal communication). Permission to access and sample orchid populations was granted by the land owners/managers for each site and collecting permits were obtained.

**Table 1.** Collection site locations across four Hawaiian Islands for *Anoectochilus sandvicensis* and *Liparis hawaiensis*, along with identities and replicates (*n*) of reference species and their average δ^15^N and δ^13^C values per plot with standard deviations (s.d.) in parentheses.

location	plot	coordinates	species name	sample type	*n*	average δ^15^N (s.d.)	average δ^13^C (s.d.)
Mt. Ka'ala, Oahu	AN01	21°30′24.29′′N, 158° 8′40.46′′W	*Anoectochilus sandvicensis*	orchid	3	−0.20 (0.29)	−33.51 (0.87)
			*Vaccinium calycinum*	reference	3	−0.53 (2.92)	−34.28 (0.53)
			*Adenophorus tamariscinus*	reference	3	−4.90 (1.49)	−33.27 (1.12)
			*Dryopteris glabra*	reference	3	−5.05 (1.41)	−30.07 (0.49)
Mt. Ka'ala, Oahu	AN02	21°30′13.37′′N, 158° 8′49.51′′W	*Anoectochilus sandvicensis*	orchid	4	1.39 (0.96)	−32.35 (1.09)
			*Peperomia membranacea*	reference	3	−2.53 (1.86)	−31.36 (1.80)
			*Metrosideros polymorpha*	reference	3	−3.77 (2.70)	−32.38 (0.71)
			*Vaccinium calycinum*	reference	3	−0.47 (0.89)	−31.86 (0.04)
Mt. Ka'ala, Oahu	AN03	21°30′26.27′′N, 158° 8′35.48′′W	*Anoectochilus sandvicensis*	orchid	3	0.94 (0.19)	−33.10 (0.69)
			*Metrosideros polymorpha*	reference	3	−3.80 (0.95)	−33.77 (0.41)
			*Coprosma granadensis*	reference	3	−1.86 (1.45)	−32.84 (0.82)
			*Dryopteris glabra*	reference	2	−1.42 (1.66)	−31.86 (0.04)
Mt. Ka'ala, Oahu	LI01	21°30′11.34′′N, 158° 8′52.61′′W	*Liparis hawaiensis*	orchid	1	−0.84	−35.39
			*Vaccinium calycinum*	reference	3	0.40 (0.40)	−34.02 (0.35)
			*Peperomia membranacea*	reference	3	−0.99 (0.46)	−30.69 (1.54)
			*Metrosideros polymorpha*	reference	3	−0.87 (1.37)	−32.58 (0.77)
Mt. Ka'ala, Oahu	LI02	21°30′11.34′′N, 158° 8′52.61′′W	*Liparis hawaiensis*	orchid	1	0.77	−32.88
			*Metrosideros polymorpha*	reference	2	−2.40 (0.57)	−32.11 (0.02)
			*Peperomia membranacea*	reference	3	−0.30 (1.48)	−30.27 (0.78)
			*Coprosma granadensis*	reference	3	−2.30 (0.69)	−29.01 (1.53)
Mt. Ka'ala, Oahu	LI03	21°30′11.73′′N, 158° 8′51.77′′W	*Liparis hawaiensis*	orchid	1	−0.01	−32.95
			*Coprosma granadensis*	reference	3	−2.22 (0.64)	−30.85 (0.77)
			*Adenophorus tamariscinus*	reference	3	−3.05 (1.61)	−33.60 (0.35)
			*Diplazium sandwichianum*	reference	3	0.02 (0.76)	−31.53 (0.40)
Waikamoi Nature Preserve, Maui	AN04	20°48′5.29′′N, 156°15′14.98′′W	*Anoectochilus sandvicensis*	orchid	1	6.55	−34.99
			*Alyxia oliviformis*	reference	3	2.36 (0.60)	−33.71 (0.19)
			*Rubus argutus*	reference	2	3.01 (2.08)	−32.89 (0.88)
			*Coprosma foliosa*	reference	2	3.48 (0.29)	−33.75 (0.24)
			*Diplazium sandwichianum*	reference	3	3.59 (0.55)	−29.69 (0.79)
Waikamoi Nature Preserve, Maui	AN05	20°47′57.88′′N, 156°15′10.54′′W	*Anoectochilus sandvicensis*	orchid	1	5.09	−35.65
			*Coprosma foliosa*	reference	3	1.51 (0.95)	−32.83 (1.04)
			*Diplazium sandwichianum*	reference	3	1.22 (0.45)	−30.16 (0.69)
			*Alyxia oliviformis*	reference	3	2.00 (0.68)	−32.96 (0.84)
Waikamoi Nature Preserve, Maui	AN06	20°47′55.05′′N, 156°15′7.24′′W	*Anoectochilus sandvicensis*	orchid	1	5.17	−35.76
			*Diplazium sandwichianum*	reference	3	2.31 (0.31)	−27.91 (0.44)
			*Rubus argutus*	reference	2	2.67 (1.12)	−33.55 (0.60)
			*Coprosma foliosa*	reference	3	3.03 (0.97)	−34.03 (0.72)
Waikamoi Nature Preserve, Maui	AN07	20°48′6.64′′N, 156°15′9.00′′W	*Anoectochilus sandvicensis*	orchid	1	5.23	−34.73
			*Alyxia oliviformis*	reference	3	1.68 (0.10)	−32.88 (0.10)
			*Diplazium sandwichianum*	reference	3	1.47 (0.72)	−30.06 (1.20)
			*Coprosma foliosa*	reference	3	1.59 (0.47)	−32.41 (1.24)
Kahili Ridge, Kauai	AN08	21°58′28.80′′N, 159°29′41.68′′W	*Anoectochilus sandvicensis*	orchid	1	2.64	−32.70
			*Bidens* sp.	reference	3	0.63 (0.99)	−32.75 (0.23)
			*Clidemia hirta*	reference	2	1.38 (0.37)	−28.79 (1.22)
			*Metrosideros polymorpha*	reference	3	−1.73 (1.53)	−32.15 (0.42)
Kahili Ridge, Kauai	AN09	21°58′29.00′′N, 159°29′41.13′′W	*Anoectochilus sandvicensis*	orchid	1	1.53	−28.38
			*Peperomia* sp.	reference	3	−2.28 (1.96)	−30.39 (0.46)
			*Clidemia hirta*	reference	3	1.23 (0.93)	−28.02 (1.20)
			*Machaerina* sp.	reference	3	2.83 (0.49)	−29.13 (1.34)
Kahili Ridge, Kauai	AN10	21°58′29.00′′N, 159°29′42.10′′W	*Anoectochilus sandvicensis*	orchid	1	4.40	−31.32
			*Pterolepis glomerata*	reference	2	1.32 (0.03)	−31.01 (1.57)
			*Melastomataceae* sp.	reference	1	1.15	−32.35
			*Clidemia hirta*	reference	3	1.40 (0.23)	−28.57 (0.85)
			*Crassocephalum* sp.	reference	3	−0.06 (0.97)	−33.68 (0.25)
Volcano National Park, Hawaii	AN11	19°27′48.24′′N, 155°14′16.26′′W	*Anoectochilus sandvicensis*	orchid	1	1.51	−35.30
			*Hedychium gardnerianum*	reference	3	−0.54 (0.92)	−32.55 (0.37)
			*Perrottetia sandwicensis*	reference	3	0.02 (0.97)	−31.90 (1.55)
			*Athyrium microphyllum*	reference	2	−4.10 (0.74)	−31.10 (0.87)
			*Metrosideros polymorpha*	reference	3	−3.75 (3.10)	−33.90 (0.31)
			*Clermontia parviflora*	reference	3	−3.08 (2.59)	−34.07 (0.38)

Vegetation types for orchid collection sites were montane wet-mesic native forests (Mt. Ka'ala, Oahu; Volcano National Park, Hawaii; Waikamoi Nature Preserve, Maui; Kahili Ridge, Kauai). I selected my sampling sites based on the following criteria: at least one cluster or individual of *A. sandvicensis* or *L.hawaiensis* was growing terrestrially within 0.5 m of at least two other understorey species, with at least two individuals per species. Owing to the rhizomatous nature of *A. sandvicensis*, determining individuals in the field is challenging. To avoid sampling the same individual twice, I separated my sampling sites by at least 10 m. I also sampled leaves of similar age and from plants of similar size in an effort to control for the influences of plant phenology on stable isotope composition. At each site, I sampled one to four leaves of *A. sandvicensis* or one leaf of *L. hawaiensis* (table 1). As site-specific references for the stable isotope composition of autotrophic plants, leaves from the surrounding understorey were also collected (table 1). To control for microsite variability that may influence the stable isotope composition of plant tissues, autotrophic leaf samples were taken from within a 0.5 m radius of their corresponding *A. sandvicensis* cluster or *L. hawaiensis* individual and from similar heights as the leaves of the orchids. Samples were bagged until I returned to either the University of Hawaii Manoa on Oahu or my lodging on the other islands where they could be further processed.

### 2.2. Stable isotope and nitrogen content analyses

Within hours of being collected, leaf samples from each individual plant were dried at approximately 80°C for a minimum of 6 h and up to 12 depending on the water content of the species. Dried leaves were ground to a fine powder, weighed and analysed for nitrogen content, nitrogen and carbon stable isotope abundances via elemental analyser/continuous flow isotope ratio mass spectrometry at the Center for Stable Isotope Biogeochemistry at the University of California Berkeley as in Hynson *et al*. [11]. Measured isotope abundances are denoted as δ-values and are calculated according to the equation: δ^15^N or δ^13^C = (*R*_sample_/*R*_standard _− 1) × 1000 [‰], where *R*_sample_ and *R*_standard_ are the ratios of heavy isotope to light isotope of the samples and the respective standard. The long-term precisions for δ^13^C and δ^15^N based on the laboratory's working standards (NIST 1577 bovine liver and sucrose solution) are: 0.1‰ for δ^13^C and 0.2‰ for δ^15^N.

### 2.3. Data analysis and statistics

**Figure 1.**
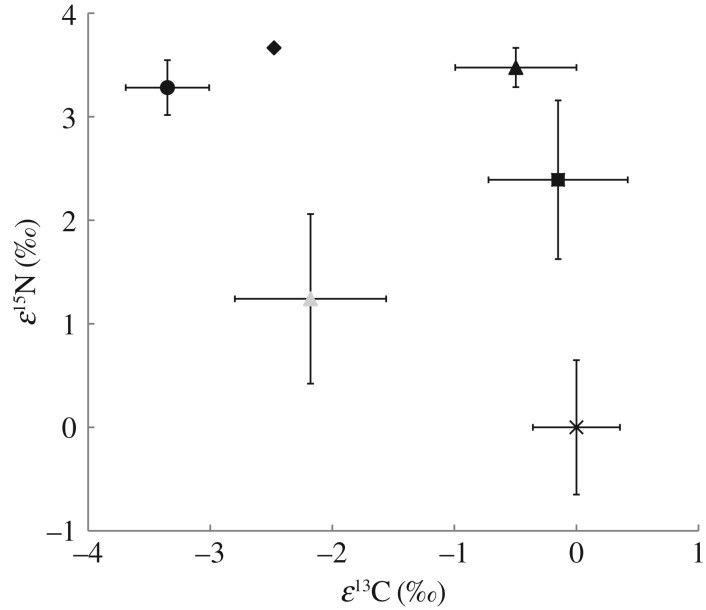
Carbon and nitrogen enrichment factors (*ε*^13^C and *ε*^15^N) for *Liparis hawaiensis* from Oahu (grey triangle), *Anoectochilus sandvicensis* from Kauai (black square), Oahu (black triangle), Hawaii (black diamond) and Maui (black circle) as well as autotrophic reference species from all sites (black X). Error bars represent 1 s.e. of the mean and in the case of autotrophic references, the largest variation in *ε*^13^C and *ε*^15^N values from all sites.

**Figure 2.**
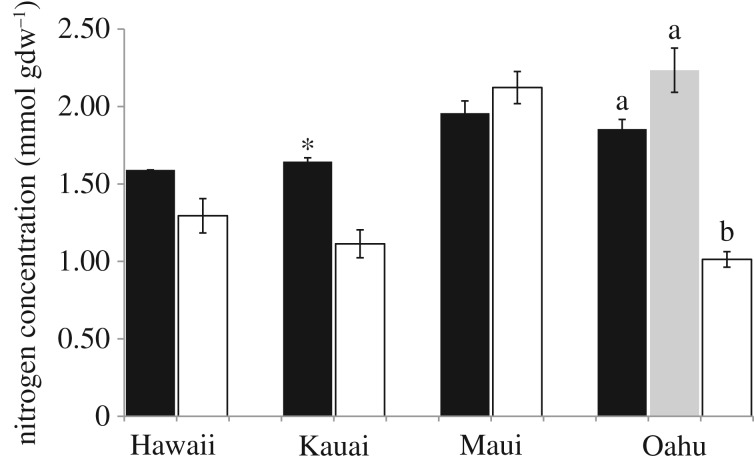
Nitrogen concentrations (mmol gdw^−1^) of the orchids *Anoectochilus sandvicensis* (black bars) across Islands and *Liparis hawaiensis* (grey bar) from Oahu relative to autotrophic references collected at the same site (open bars). Asterisk represents marginally statistically significant differences between *A. sandvicensis* and references, while different letters represent statistically significant differences at *α* ≤ 0.05. Error bars represent one standard error of the mean.

Because leaf stable isotope compositions are influenced by local climatic conditions, to make comparisons of these values among islands I used a data normalizing calculation know as an isotope enrichment factor (*ε*) approach [37]. Enrichment factors were calculated on a site-by-site basis in the following way: *ε* = δ*X*_sample_ − δ*X*_ref_, where δ*X*_sample_ is the δ^15^N or δ^13^C of an individual sample (*A. sandvicensis*, *L. hawaiensis* or autotrophic species) and δ*X*_ref_ is the mean δ^15^N or δ^13^C of all autotrophic plants from a given site. Enrichment factors per island and species (or group in the case of autotrophs) were then averaged (figure 1). Average nitrogen (N) concentration (mmol gdw^−1^) by orchid species and autotrophic references per island are shown in figure 2. All values are shown ± 1 s.e.

Shapiro–Wilk tests revealed that none of the data were consistently normally distributed for N concentration, *ε*^13^C or *ε*^15^N, thus statistical comparisons between *A. sandvicensis, L. hawaiensis* and autotrophic references for all three islands were made using non-parametric Kruskal–Wallis tests with Bonferroni corrections for multiple comparisons when appropriate. Statistical comparisons could not be made for Hawaii Island due to inadequate replication (*A. sandvicensis n* = 1). Tests incorporated the spread of enrichment factors not only for *A. sandvicensis* and *L. hawaiensis*, but the autotrophic references as well since individual replicates of reference species are also subjected to the enrichment factor calculation (i.e. statistical comparisons of the two orchid species are not to zero, but the variation of the references from zero). Comparisons of the nitrogen concentrations, δ^15^N, *ε*^15^N and *ε*^13^C between *L. hawaiensis* and its temperate relatives *Liparis nervosa* and *L. loesellii* were made using a Kruskal–Wallis test due to non-normal distribution of the data. Comparisons of the same factors for *A. sandvicensis* and its temperate relatives in subtribe Goodyerinae (*Goodyera repens, G. oblongifolia, G. schlechtendaliana* and *Zeuxine agyokuana*) were also made using a Kruskal–Wallis test. Data on the temperate relatives of *L.hawaiensis* and *A. sandvicensis* were extracted from prior publications [11,18,38] and in the case of *L.loesellii*, Julienne Schiebold 2016, personal communication. All statistics were carried out in SPSS v. 24 (IBM Armonk, NY, USA) and considered significant at *α* ≤ 0.05.

## 3. Results

Delta ^13^C values of all plants sampled in this study were within the known ranges for C3 species (table 1) [39]. Patterns of ^15^N and ^13^C composition and leaf nitrogen concentrations for *A. sandvicensis* and *L. hawiensis* shared some similarities with their temperate relatives, but were also species and location specific (figures 1 and 2; table 2). *Anoectochilus sandvicensis* was consistently enriched in ^15^N relative to surrounding vegetation (Kauai mean *ε*^15^N 2.39(0.77)‰, *p* = 0.018; Maui mean *ε*^15^N 3.28(0.26)‰, *p* = 0.001, Oahu mean *ε*^15^N 3.47(0.19)‰, *p* < 0.001; figure 1). Patterns of ^13^C composition in *A. sandvicensis* relative to surrounding vegetation among islands were less consistent. On Kauai and Oahu, *A. sandvicensis* *ε*^13^C values were not significantly different from those of reference plants (Kauai mean *ε*^13^C −0.15(0.57)‰, Oahu mean *ε*^13^C −0.50(0.31)‰; figure 1), while on Maui, *A. sandvicensis* was significantly depleted in ^13^C relative to references (mean *ε*^13^C −3.35(0.34)‰, *p* = 0.002; figure 1). While statistical tests were not appropriate for comparisons of the single Hawaii Island individual of *A. sandvicensis*, it was depleted in ^13^C (*ε*^13^C −2.48‰) and enriched in ^15^N (*ε*^15^N 3.67‰) relative to references (figure 1). *Anoectochilus sandvicensis* had higher leaf N concentrations relative to references on all the islands except Maui, but only on Oahu were differences in leaf N concentrations statistically significant (*p* < 0.001; figure 2), while marginally so on Kauai (*p* = 0.06; figure 2).

**Table 2.** Comparisons of the tropical endemic orchids *Anoectochilus sandvicensis* and *Liparis hawaiensis* mean nitrogen concentrations (mmol gdw-1), δ^15^N, *ε*^13^C and *ε*^15^N values to related temperate taxa. Parentheses in the first column contain number of replicates (*n*), while in the following columns are one standard error of the mean (s.e.). Asterisks represent statistically significant differences between tropical and temperate orchids for a given factor.

orchid (*n*)	mean nitrogen concentration mmol gdw^−1^ (s.e.)	mean δ^15^N‰ (s.e.)	mean *ε*^15^N‰ (s.e.)	mean *ε*^13^C‰ (s.e.)
*Anoectochilus sandvicensis* (18)	1.82 (0.05)*	2.23 (0.51)*	3.26 (0.19)*	−1.18 (0.36)*
temperate Goodyerinae (21)	1.43 (0.16)	−3.24 (0.43)	1.66 (0.27)	−2.6 (0.52)
*Liparis hawaiensis* (3)	2.24 (0.14)	−0.3 (0.46)	1.24 (0.82)	−2.17 (0.62)
temperate *Liparis* spp. (8)	2.07 (0.17)	−0.53 (0.56)	2.76 (0.57)	−0.93 (0.92)

*Liparis hawaiensis* from Mount Ka'ala, Oahu was not significantly enriched in ^15^N (mean *ε*^15^N 1.24(0.82)‰) relative to surrounding references while it was significantly depleted in ^13^C (mean *ε*^13^C −2.18(0.62)‰, *p* = 0.047; figure 1), and had significantly greater leaf N concentrations than reference species (*p* = 0.003; figure 1). Average leaf N concentrations for *L. hawaiensis* were the highest among our test species and significantly higher than references (*p* = 0.003; figure 2). Nitrogen concentrations of *L. hawaiensis* were not significantly different from *A. sandvicensis* from the same location (figure 2). Concurrently, *ε*^13^C and *ε*^15^N values were statistically indistinguishable between *A. sandvicensis* and *L.hawaiensis* individuals from Oahu. However, *A. sandvicensis* was on average more enriched in ^15^N and ^13^C than *L. hawaiensis* (figure 1).

Nitrogen concentrations and δ^15^N values for *A. sandvicensis* from across all islands were significantly higher than its temperate relatives in Goodyerinae (*p* = 0.002 and *p* < 0.001, respectively; table 2). Similarly, *ε*^15^N and *ε*^13^C values of *A. sandvicensis* were significantly higher than its temperate counterparts (*p* ≤ 0.001 and *p* = 0.041, respectively; table 2). Conversely, while on average *L. hawaiensis* had higher N concentrations and δ^15^N values relative to its temperate congeners, its *ε*^15^N and *ε*^13^C values were more depleted and none of these values were statistically distinguished from their temperate counterparts (table 2).

## 4. Discussion

Tropical terrestrial orchid species are numerous, but to date there has been a bias in the literature towards examining the ecophysiologies of temperate species [6]. To address this gap, I provide the first data on the carbon and nitrogen ecophysiologies of two out of the three orchid species endemic to Hawaii. I found that among islands, populations of *A. sandvicensis* were consistently enriched in ^15^N relative to surrounding vegetation. The on-average 3.05‰ ^15^N enrichment in *A. sandvicensis* is biologically significant. For example, it is greater than differences in ^15^N relative abundance between congeneric and sympatric tropical plant species that either derive N from ant debris or the soil [40], and greater than differences among conspecifics of an insectivorous plant species where some individuals derive a greater portion of N from litterfall than prey [41]. Furthermore, the ^15^N enrichment found in *A. sandvicensis* is similar to other temperate individuals of Goodyerinae, but even more pronounced (table 2). Along with significant relative enrichment in ^15^N, *A. sandvicensis* generally also had higher N concentrations than surrounding vegetation. However, N concentrations in *A. sandvicensis* were only significantly higher than references species on Oahu, and were on average, lower among Maui individuals. Similar to patterns of ^15^N enrichment, N concentrations among all *A. sandvicensis* individuals were significantly higher than those of its temperate relatives (table 2).

The lines of evidence supporting partial mycoheterotrophy among rhizoctonia-associated orchids are mounting. First, all orchids studied to date are initially mycoheterotrophic, thus the physiology to feed off of fungi exists [6]. Second, Cameron *et al*. [42] found transfer of carbon from rhizoctonias to adult *Goodyera repens ex situ*, indicating that mycoheterotrophic C-gains can persist past germination [42]. Third, the results of Gebauer *et al*. [10] demonstrated similarities in the isotope profiles of fully and partially mycoheterotrophic ectomycorrhizal orchids relying on fungal-derived substrates and rhizoctonia-associated orchids [10]. The current findings show that the temperate patterns of ^15^N enrichment and N content hold at least for two tropical rhizoctonia-associated orchid species and provide evidence of possible partial mycoheterotrophy. The results from *A. sandvicensis* also support the hypothesis that due to generally relatively greater N availability in the tropics, tropical terrestrial orchids can have an additive enrichment in ^15^N and N concentrations relative to temperate species.

In the case of *A. sandvicensis*, *ε*^13^C values relative to surrounding vegetation varied by sampling location (figure 1). This variation may be related to the light environment in which individual orchids were found. Liebel *et al*. [43] found that the δ^13^C values of *G. repens* (Goodyerinae) in Norway responded to increases in light availability in a similar manner as surrounding autotrophic references by becoming more ^13^C enriched as irradiance increased [43]. Indeed, both the Oahu and Kauai sites from the current study were far more exposed (mountain and ridge tops) relative to the Hawaii and Maui sites, which are deeply shaded wet-mesic tropical forests. Alternatively, the increase in *ε*^13^C values among Oahu and Kauai populations relative to Hawaii and Maui could be owed to relative increases in fungal carbon gains. Thus, based solely on *ε*^13^C the degree of partial mycoheterotrophy in *A. sandvicensis* remains uncertain. However, recent work by Gebauer *et al*. [10] has highlighted that ^13^C natural abundance may not be the best indicator for reliance on fungal-derived compounds among rhizoctonia-associated orchids.

Though from a small sample size (*n* = 3), ^15^N enrichment and N concentrations of *L. hawaiensis* are not significantly greater than temperate congeners. This indicates that there are not always consistent additive effects of a tropical climate and belonging to Orchidaceae on tropical orchid species N physiologies. Similar to the temperate species *L. nervosa* [38]*, L. hawaiensis* from Mount Ka'ala, Oahu was on average (though not significantly so) enriched in ^15^N relative to surrounding references, while similar to *L. loesellii* [44] it was significantly depleted in ^13^C (figure 1), and had significantly greater leaf N content than reference species (figure 2). Previously, *L. nervosa* has been estimated to receive upwards of 21% of its carbon and 18% of its nitrogen via mycoheterotrophy [6]. Given that *L. nervosa* and *L. hawaiensis* have similar relative ^15^N enrichment, and that *L. hawaiensis* has N concentrations within the same range as *L. nervosa* (table 2), this species is also a candidate for partial mycoheterotrophy. However, similar to *A. sandvicensis*, *ε*^13^C values alone provide no clear indication of partial mycoheterotrophy. Interestingly, the genus *Liparis* may be predisposed to the evolution of mycoheterotrophy as it belongs to subfamily Epidendroideae which contains fully mycohetertrophic taxa and there exists a leafless, but green stemmed South American species *L. aphylla* (a likely candidate for partial or full mycoheterotrophy) [6,45].

## 5. Conclusion

The current definition of mycoheterotrophy put forth by Merckx [3, p. 10] is ‘the ability of a plant to obtain carbon from fungi’. Originally, one of the most useful indicators of carbon gains via fungi in potentially mycoheterotrophic species was ^13^C enrichment above that of surrounding vegetation and more similar to the fungi on which they depend [6]. This pattern of ^13^C enrichment among partially and fully mycoheterotrophic species follows the isotope food-chain model put forth by Fry [46] and ^13^C enrichment appears to be a consistent trait among mycoheterotrophic Orchidaceae that partner with ectomycorrhizal fungi [47]. However, this is generally not the case for rhizoctonia-associated orchids, which are often depleted in ^13^C or show no difference in ^13^C enrichment relative to surrounding autotrophs [6]. This makes detection of mycoheterotrophy less straightforward in terrestrial rhizoctonia-associated orchid species and has led researchers to question whether these species maintain mycoheterotrophic C-gains post germination [11]. Some rhizoctonia-associated orchids may still be capable of mycoheterotrophy, as significant ^15^N enrichment along with other ecophysiological traits emerge as additional indicators of carbon gains via fungi [10]. Furthermore, based on the results of this study, patterns of significant ^15^N enrichment and high N content among rhizoctonia-associated orchids hold, or are even more pronounced in the tropics than temperate regions. This suggests that ^15^N enriched rhizoctonia-associated orchids should be considered an additional functional guild of mycoheterotrophs [6,11]. Future efforts should be focused on examining the ecophysiologies of additional tropical terrestrial orchid taxa, including deuterium composition, identifying the fungal partners of these species, as well as the mechanisms by which compounds are exchanged between rhizoctonias and their orchid partners.

Tropical species, especially those that are island endemics, are considered some of the most sensitive to the negative effects of global change such as habitat fragmentation and invasive species [48,49]. Therefore, they should be research and conservation priorities. However, information on the interactions of island endemic tropical plants with their microbial symbionts such as mycorrhizal fungi, which are critical for plant establishment, growth and reproduction is sorely lacking [50]. Until there are more efforts put forth to study these interactions, it remains unknown whether the ecologies and ecophysiologies of temperate and tropical species are comparable. This study highlights the parallels and differences among the ecophysiologies of tropical and temperate species for an important group of globally distributed plants—the orchids.

## Data accessibility

All isotope data analysed in this study are summarized in tables 1 and 2 including replicates of each sample, and metadata (site locations and species sampled). All nitrogen data analysed in this study are summarized in the results and figure 2, metadata for nitrogen are also included in tables 1 and 2.
